# Mechanism of Pulp Regeneration Based on Concentrated Growth Factors Regulating Cell Differentiation

**DOI:** 10.3390/bioengineering10050513

**Published:** 2023-04-25

**Authors:** Sijing Yu, Yi Zheng, Qiang Guo, Wenxu Li, Ling Ye, Bo Gao

**Affiliations:** 1 State Key Laboratory of Oral Diseases, National Clinical Research Center for Oral Diseases, Department of Cariology and Endodontics, West China Hospital of Stomatology, Sichuan University, Chengdu 610041, China; yusijing@stu.scu.edu.cn (S.Y.);; 2 State Key Laboratory of Oral Diseases, National Clinical Research Center for Oral Diseases, West China Hospital of Stomatology, Sichuan University, Chengdu 610041, China

**Keywords:** concentrated growth factors, dental pulp cells, tissue engineering, pulp regeneration therapy

## Abstract

Concentrated growth factors (CGF) is the newest generation platelet concentrate product, which has been reported to promote the proliferation and differentiation of human dental pulp cells (hDPCs). However, the effect of liquid phase of CGF (LPCGF) has not been reported. This study was aimed to evaluate the influence of LPCGF on the biological properties of hDPCs, and to explore the in vivo mechanism of dental pulp regeneration based on the hDPCs-LPCGF complex transplantation. It was found that LPCGF could promote the proliferation, migration and odontogenic differentiation of hDPCs, and 25% LPCGF induced the most mineralization nodule formation and the highest DSPP gene expression. The heterotopic transplantation of the hDPCs-LPCGF complex resulted in the formation of regenerative pulp tissue with newly formed dentin, neovascularization and nerve-like tissue. Together, these findings provide key data on the effect of LPCGF on the proliferation, migration, odontogenic/osteogenic differentiation of hDPCs, and the in vivo mechanism of hDPCs-LPCGF complex autologous transplantation in pulp regeneration therapy.

## 1. Introduction

Caries, odontodysplasia and dental trauma can cause pulp necrosis and affect the root development of young permanent teeth. Due to the wide apical foramen and root canal, and the thin dentin wall easily to be fractured, the root canal preparation and filling in young permanent teeth are quite challenging. How to promote the recovery of the pulp health of young permanent teeth and maintain the continuous development of the root is an important challenge in the current endodontic therapy [1,2].

Regenerative endodontic therapies provide a method to replace diseased pulp tissue with new regenerated pulp tissue based on regenerative medicine and biological tissue engineering technology [3,4,5]. From the perspective of application, pulp regeneration therapy includes pulp revascularization, autologous pulp stem cell replantation and cell homing technique [6]. Human dental pulp stem cells (hDPSCs) are a type of stem cells isolated from pulp tissue, which have multipotential differentiation ability. These cells are important source of stem cells to achieve the repair and reconstruction of pulp tissue and have the potential to form pulp-dentin complex [7,8]. Studies have demonstrated that pulp stem cell transplantation can regenerate pulp-like tissue, including the regeneration of pulp-like tissue in ectopic tooth transplantation models [9,10], as well as the clinical observation of healing of apical lesions, restoration of pulp vitality [11], and histological observation of formation of mineralized fibrous connective tissue similar to pulp-like tissue in the root canal [12,13,14].

Growth factors play a vital role in cell proliferation, migration, differentiation and angiogenesis. Autologous platelet concentrates which contain high levels of growth factors are widely applied in dental pulp regeneration research [15,16,17,18]. Different types of autologous platelet concentrates, including platelet rich plasma (PRP), platelet rich fibrin (PRF) and concentrated growth factor (CGF), can be obtained by the exact method of centrifugation. PRP, as the first generation of autologous platelet concentrates, is prepared by the centrifugation of autologous whole blood together with thrombin and calcium chloride [19]. Due to the addition of exogeneic thrombin and anticoagulant in the preparation process, this technology might cause an immunologic and infectious response, making its use controversial [19,20]. In addition, activation with thrombin prior to implantation has been shown to significantly inhibit osteoinductive activity of PRP [21]. The preparation of PRF does not require the addition of any exogenous material and is more widely used in oral and maxillofacial surgical procedures [22]. CGF is the newest platelet concentrate and is produced in a manner similar to that used to produce PRF but involves different centrifugation speeds. There are significant differences in the amounts of growth factors in PRP, PRF and CGF [23]. PRF and CGF extracts seem to contain compatible or higher levels of platelets and platelet-derived growth factors compared to PRP preparations [24]. Studies have shown that the growth factors in CGF can accelerate the process of wound healing and promote tissue regeneration by stimulating cell proliferation and differentiation and promoting stem cell migration, and CGF has been widely used in clinical practice to promote bone tissue formation and soft tissue healing [24,25,26].

Currently, in vitro study has shown that CGF can promote the proliferation and migration of umbilical vein endothelial cells and the formation of tubular structures [27,28], and another in vitro study has also shown that CGF has a positive effect on the proliferation, migration and differentiation of hDPSCs stimulated by lipopolysaccharide [29]. In potential cell-based tissue engineering, LPCGF is more likely to fully mix and contact with stem cells than CGF, providing stem cells with an extracellular microenvironment that is more conducive to pulp tissue regeneration. However, there have been no reports on the role of LPCGF on promoting hDPCs to form pulp-dentin complex in vivo currently. In our study, we hypothesized that LPCGF contains significant growth factors and might promote pulp tissue regeneration. Then we explored the release and degradation trend of growth factors in autologous CGF and LPCGF, the role of LPCGF on proliferation, migration and differentiation of hDPCs in vitro, and on promoting the formation of heterotopic pulp-like tissue in vivo, so as to provide the mechanism for the clinical application of autologous transplanted hDPCs-LPCGF complex in regenerative endodontic therapy.

## 2. Materials and Methods

### 2.1. Isolation and Culture of hDPCs

Healthy, intact and caries-free third molars were collected from 18–22 years old patients in the Oral Surgery Department of West China Hospital of Stomatology of Sichuan University, with informed consent of the patients. The hDPCs were isolated by enzyme digestion according to a previously described method [30]. The separated cells were cultured in Dulbecco’s Modified Eagle’s Medium (Corning, NY, USA), supplemented with 10% fetal bovine serum (FBS; Gibco, Billings, MT, USA) and 100 U/mL Penicillin-Streptomycin (PS; Hyclone, Logan, UT, USA) in a humidified atmosphere of 5% CO_2_ at 37 °C. Cells at passage 3 (P3) were used in the following study.

### 2.2. Conditioned Medium Preparation

This study has been approved by the Medical Ethics Committee of West China College of Stomatology, Sichuan University (Ethics Approval Number: WCHSIRB-D2017-215). The volunteers were aged 18–25 years old, and were confirmed that they did not have any of the following conditions: (1) infectious diseases; (2) blood diseases; (3) history of infection and taking antibiotics in the last 3 months; (4) taking aspirin and other drugs that affect platelet function in the last 3 months; (5) women in pregnancy, lactation and menstruation.

#### 2.2.1. Preparation of CGF Extract

Venous blood were collected using red tube cap samplers (no additives) and green tube cap samplers (containing heparin sodium anticoagulant). Then the tubes were immediately centrifuged in the MediFuge centrifuge (Silfradent, Santa Sofia, Italy) by 30 s acceleration, 2 min at 2700 rpm (600 g), 4 min at 2400 rpm (400 g), 4 min at 2700 rpm (600 g), 3 min at 3000 rpm, and 36 s deceleration [31].

The CGF gel (middle layer) in the red cap tube was washed with sterile PBS, frozen with liquid nitrogen for 5 min, and melted at 37 °C for 5 min, repeated three times. Then the CGF extract was centrifuged and filtered for subsequent ELISA experiment. The LPCGF (middle layer) in the green cap tube was divided into three parts. The first part was directly prepared for CGF conditional medium, the second part was heated by activated plasma albumin gel (APAG) device at 75 °C for 5 min for ELISA experiment, co-culture experiment with hDPCs and transplantation of hDPCs-LPCGF complex in nude mice in vivo.

#### 2.2.2. Preparation of CGF Conditional Medium

Each 1 mL LPCGF was added into 9 mL blank medium for 100% LPCGF. The 100% LPCGF was further diluted with blank medium to obtain 50% and 25% LPCGF. Conditioned media containing three concentrations of LPCGF were completed after being supplemented with 10% FBS and 100 U/mL PS.

### 2.3. Scanning Electron Microscopy (SEM)

LPCGF and hDPC suspension were mixed and evenly inoculated on the cell sliders for co-culture. After 7 days, the cell slides were fixed with 2.5% glutaraldehyde, dehydrated with ethanol gradient, and dried at critical point. Then cell slides were sprayed with gold coating, and the adhesion of hDPCs to LPCGF fibrin network was observed by SEM (JSM-7500F, Tokyo, Japan).

### 2.4. Cell Proliferation Assay

The hDPCs were seeded on 96-well plates (Corning, NY, USA) at a density of 2 × 10^3^ cells per well. The cells were divided into control group, 25% LPCGF, 50% LPCGF and 100% LPCGF groups. Cell Counting Kit-8 (Beyotime, Shanghai, China) was used to analyze the cell numbers.

### 2.5. Cell Migration Assay

The hDPCs were divided into control group (normal medium without FBS), 25%, 50% and 100% LPCGF groups (without FBS), and 2% FBS group. After 1 day of culture, a sterile tip was used to scrape horizontal lines at the bottom of each well. After being observed and photographed using an inverted microscope (Olympus, TH4-200, Tokyo, Japan), the cells were cultured for 12 h and then photographed again.

### 2.6. Alizarin Red S Staining

The cells were divided into four groups: control group, namely mineralization induction solution group (normal medium containing 10% FBS and 100 U/mL PS added with 10 nmol/L dethasone, 10 mmol/L N-glycerophosphate sodium and 50 mg/L vitamin C), and mineralization induction solution added with 100% LPCGF, 50% LPCGF and 25% LPCGF groups. After 7 and 14 days, the hDPCs were fixed in 4% paraformaldehyde, stained with 0.1% alizarin red S solution (Solarbio, Beijing, China), and the formation of mineralization nodules was photographed.

### 2.7. Detection of ALP Activity

The grouping was the same as Alizarin red S staining. After 7 days, the hDPCs were fixed in 4% paraformaldehyde, added with alkaline phosphatase staining solution (Beyotime, Shanghai, China), and incubated at room temperature for 30 min. Then the formation of mineralization nodules was observed and photographed.

### 2.8. Real-Time Quantitative Polymerase Chain Reaction

The mRNA expression levels of dentin sialophosphoprotein (DSPP), Runt-related transcription factor 2 (Runx2) were determined by quantitative polymerase chain reaction (qPCR). Total RNA was isolated from cells using TRIZOL reagent. Then, 1μg of total RNA was reversely transcribed into cDNA using PrimeScript™ Kit (Takara, Maebeshi, Japan). The cDNA samples were then amplified using SYBR Premix Ex TaqTM Kit (Takara, Maebeshi, Japan) according to the manufacturer’s instructions. The relative mRNA expression was determined using the comparative Ct (^ΔΔ^Ct) method. The primer sequences for DSPP and Runx2 are shown in Table 1.

### 2.9. Growth Factor Detection by Enzyme-Linked Immunosorbent Assay (ELISA)

The contents of VEGF, BMP2, PDGF-BB and bFGF were determined quantitatively by enzyme-linked immunosorbent assay (ELISA) at 1 and 7 days. The ELISA kit (SunRedBio, Shanghai, China) was used according to the manufacturer’s instructions.

### 2.10. In Vivo Transplantation Assay of hDPCs and Concentrated Growth Complex in Nude Mice

The 6-week-old female nude mice were obtained from Experimental Animal Center of Sichuan University. The tooth transplantation models were prepared similar to a previously described method [10]. Eighteen single-rooted teeth were randomly divided into three groups. The root canal was performed to the size of 04/35 with MTwo nickel-titanium instruments (VDW, Munchen, Germany). Then the root part was prepared into an 8 mm-long root model with an opening apical orifice of 2 mm in diameter, and alternately rinsed and vibrated with 1.25% NaClO and 17% EDTA solutions. After autoclave sterilization, the coronal orifice of the root models was sealed with MTA and glass ionomer cement.

The hDPCs were resuspend with a density of 1 × 10^5^ cells/mL using normal medium. The gelatinous LPCGF was mixed with the same volume of cell suspension through a three-way tube. The hDPCs-LPCGF complex or hDPCs suspension were separately injected into different root models, and then apical openings were sealed with absorbable gelatin sponge. Different groups of root models were implanted into the subcutaneous tissue on the back of the nude mice. After two months, the subcutaneously transplanted root models were collected for Micro-CT analysis, hematoxylin-eosin (HE) staining and immunohistochemistry (IHC).

### 2.11. Micro-CT Analysis

Before the in vivo transplantation, the prepared root models were scanned at a voxel size of 10.0 μm in a microcomputer tomography unit (SCANCO Medical AG, CH-8306 Bruettisellen, Switzerland) at settings of 70 kVp and 200 μA. Two months after the operation, the transplanted root models of each group were scanned with the same parameters. The total volume (TV, including the medullary cavity) and the hard tissue volume (bone volume, BV) of the root models were calculated using data analysis software (SCANCO Medical Evaluation), and the BV/TV values of each root model were obtained. Then the ΔBV/TV values were obtained to indicate the changes in hard tissue volume of the root models after the operation, so as to evaluate whether new mineralization tissue was formed.

### 2.12. Hematoxylin-Eosin Staining

The postoperative samples were demineralized in EDTA solution and prepared into sections. Then the sections were deparaffinized, rehydrated, and then stained by HE staining reagent according to the manufacturer’s instructions.

### 2.13. IHC

The primary antibodies used for immunochemistry were DSPP, NESTIN and VEGF (Immunoway, Plano, TX, USA). Secondary antibodies were all purchased from ZenBioScience. The sections were deparaffinized, followed by incubation in trypsin antigen retrieval buffer, sealed with oxidoreductase to suppress endogenous peroxidase activity, and incubated with primary antibody at 4 °C overnight. Then the sections were incubated with secondary antibody for 30 min at 37 °C. The sections were added with color developing agent DAB (DAKO, Glostrup, Denmark) and counterstained with hematoxylin.

### 2.14. Statistical Analysis

Statistical analysis was performed by using SPSS version 19.0 statistical software. The one-way analysis of variance (ANOVA) test and Tukey’s *t*-test were used for statistical analysis. Statistical significance was accepted at *p* < 0.05.

## 3. Results

### 3.1. Characteristics of hDPCs

**Figure 1:**
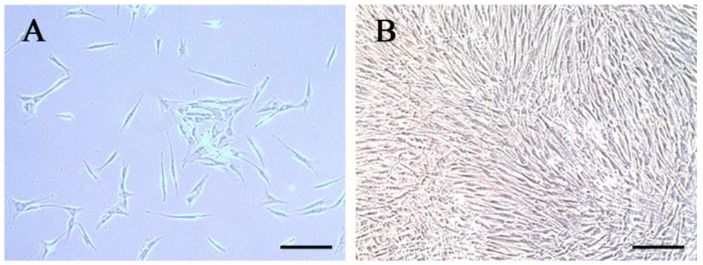
The characteristics of hDPCs: (**A**) The primary hDPCs; (**B**) the cells after passage. Scale bar = 200 μm.

The primary hDPCs exhibited a spindle shape with a single nucleus, and some cells were elliptic or polygonal in scattered distribution. After passage, the hDPCs were mostly spindle-shaped and closely arranged (Figure 1), and multi-layer growth could be observed after single layer overgrowth.

### 3.2. Adhesion of hDPCs to LPCGF Fibrin Network

**Figure 2:**
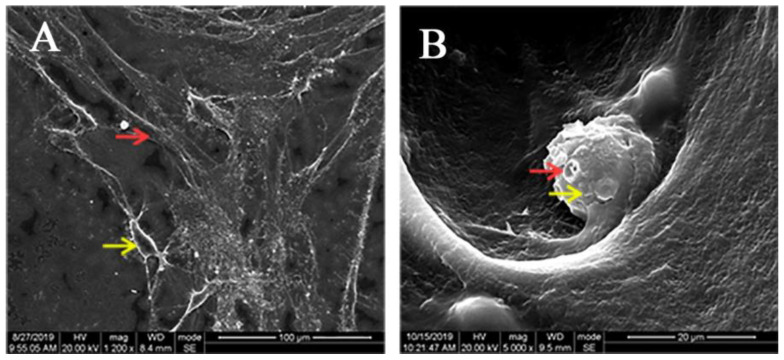
Adhesion of hDPCS to LPCGF fibrin network. (**A**) Yellow arrowhead indicates hDPCs, and red arrowhead indicates fibrin network; (**B**) Yellow arrowhead indicates white blood cells, and red arrowhead indicates red blood cells.

After the co-culture of hDPCs and LPCGF for 7 days, hDPCs were growing in a relatively large number and dense distribution. The cells extended many long and thin pseudopods connected to each other to form a laminated network. White blood cells (large and spherical) and red blood cells (disc-shaped and double-concave) were scattered in the interspaces of LPCGF fibrin network (Figure 2).

### 3.3. Effect of LPCGF on hDPC Proliferation

**Figure 3:**
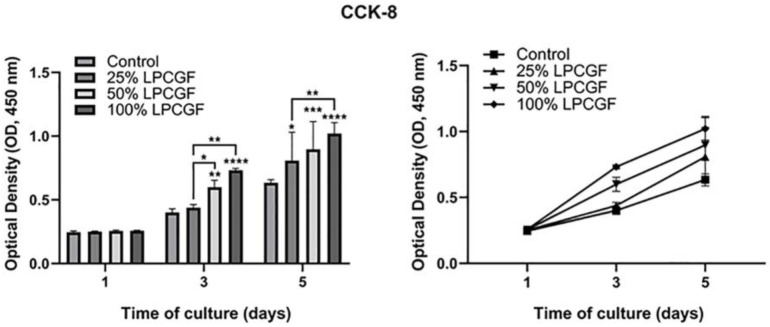
Effect of LPCGF on hDPC proliferation for 1, 3 and 5 days. (* *p* < 0.05, ** *p* < 0.01, *** *p* < 0.001, **** *p* < 0.0001).

100%, 50% and 25% LPCGF showed no accelerated effect on hDPC proliferation on day 1 (*p* > 0.05), and all increased proliferation rate of hDPCs on day 5 (*p* < 0.05, *p* < 0.001 and *p* < 0.0001). Different concentrations of LPCGF could increase cell proliferation in a dose-dependent manner, and 100% LPCGF showed the most obvious promoting effect on the proliferation of hDPCs (Figure 3).

### 3.4. Effect of LPCGF on hDPC Migration

**Figure 4:**
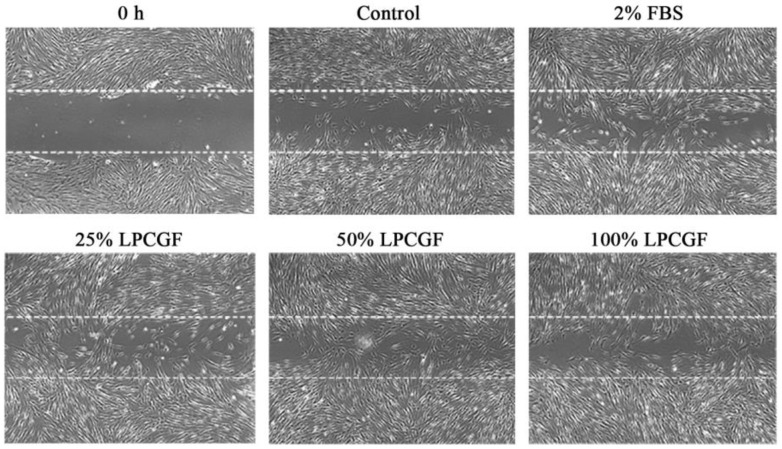
Effect of LPCGF on hDPC migration for 12 h.

The effect of 25% and 50% LPCGF on cell migration was greater than that of 100% LPCGF group, and all LPCGF groups showed higher migration ability than that of control group (Figure 4).

### 3.5. The Effect of LPCGF on hDPC Differentiation

**Figure 5:**
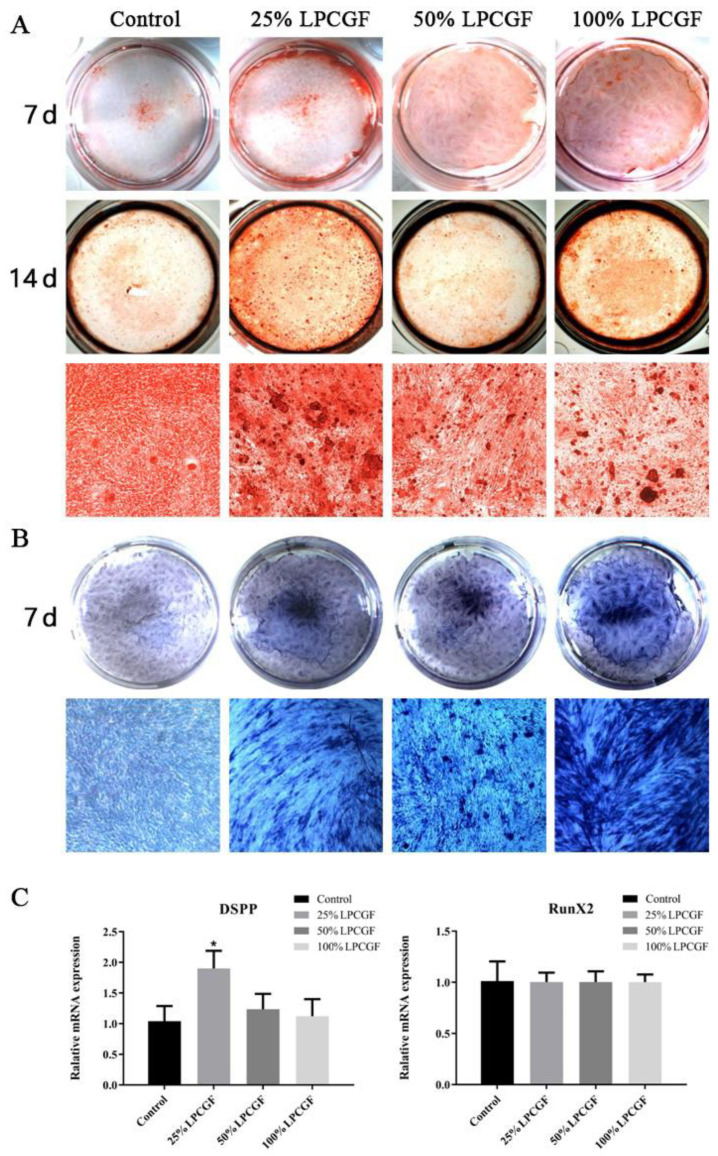
The effect of LPCGF on hDPC mineralization and differentiation. (**A**) The effects of LPCGF on alizarin red staining in hDPCs after culture for 7 days and 14 days. (**B**) The effects of LPCGF on ALP staining in hDPCs after culture for 7 days. (**C**) Relative gene expression levels of DSPP and Runx2 of the hDPCs after culture for 7 days. (* *p* < 0.05).

The results of alizarin red S staining (Figure 5A) showed that the osteogenic induction medium with different concentrations of LPCGF could successfully induce osteogenic differentiation and mineralization nodule formation of hDPCs. On day 14, the number and area of mineralization nodules in the LPCGF groups were increased. In particular, the number and area of mineralization nodules in the 25% LPCGF group were higher than those in the other two LPCGF groups. The results of ALP staining (Figure 5B) showed that the LPCGF groups with different concentrations had deeper staining than the control group, indicating that LPCGF could promote the odontogenic differentiation of hDPCs.

DSPP and Runx2 expression in hDPCs after 7 days of mineralization induction were shown (Figure 5C). DSPP expression in the 25% LPCGF group was 1.8-fold higher than that in the control group (*p* < 0.05), while Runx2 expression in the LPCGF groups with different concentrations had no significant difference from that in the control group (*p* > 0.05).

### 3.6. Changes of Growth Factor Content in CGF and LPCGF

**Figure 6:**
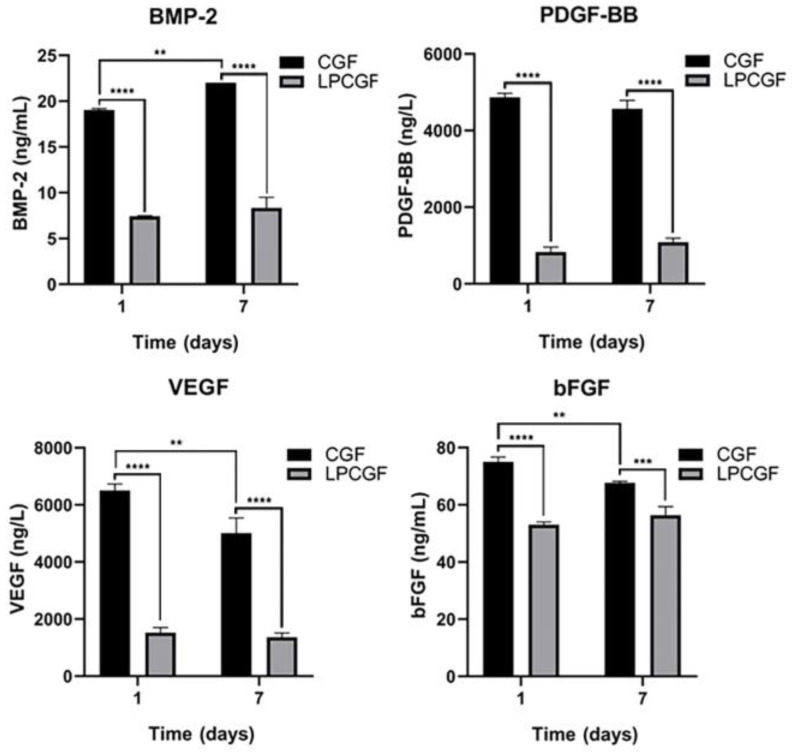
The concentrations of bFGF, BMP-2, PDGF and VEGE in CGF and LPCGF. (** *p* < 0.01, *** *p* < 0.001, **** *p* < 0.0001).

The concentrations of bFGF, BMP-2, PDGF and VEGF in the CGF group were higher than those in the heat-treated LPCGF group (*p* < 0.05). Over time, the BMP-2 release in CGF group showed an upward trend (*p* < 0.01), while VEGF and bFGF showed a downward trend (*p* < 0.01), and PDGF-BB showed no significant change. In the LPCGF group, the release of these four growth factors was relatively stable, and no significant changes were observed with time (Figure 6).

### 3.7. The Effect of hDPCs-LPCGF Complex on the Pulp Tissue Regeneration and the Root/Apical Foramen Development In Vivo

**Figure 7:**
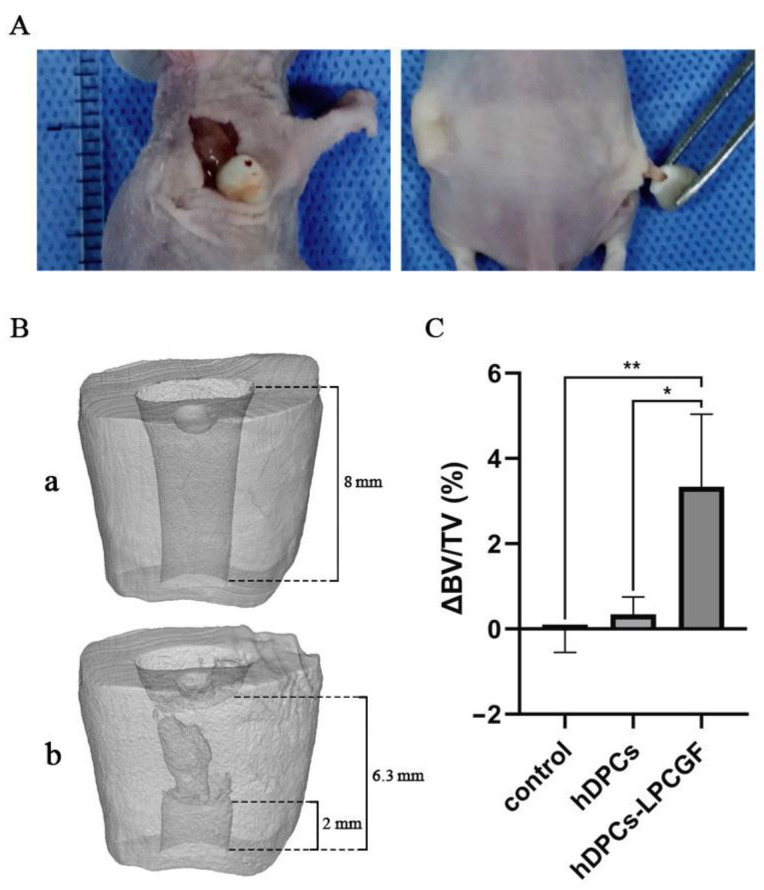
The gross and micro-CT results of hDPCs-LPCGF complex on the pulp tissue regeneration in vivo. (**A**) New soft tissues were visible in the root canal of the root models after 2 months of hDPCs-LPCGF transplantation. (**B**) The preoperative (**a**) and postoperative (**b**) cross sections of the root models in hDPCs-LPCGF complex group. (**C**) The increase of hard tissue in hDPCs-LPCGF, hDPCs and control groups. (* *p* < 0.05, ** *p* < 0.01).

Two months after the transplantation, the nude mice were sacrificed, and new soft tissues were visible in the root canal of the root models (Figure 7A).

#### 3.7.1. Micro-CT Results

Newly formed hard tissue could be observed in the root canal wall of the root models in the hDPCs-LPCGF complex group (Figure 7B), while no obvious new hard tissue was observed in the control group and hDPCs group. Micro-CT analysis showed that the increase of hard tissue in hDPCs-LPCGF group was significantly higher than that in control group and hDPCs group (*p* < 0.01 and *p* < 0.05) (Figure 7C).

#### 3.7.2. Histologic Results

**Figure 8:**
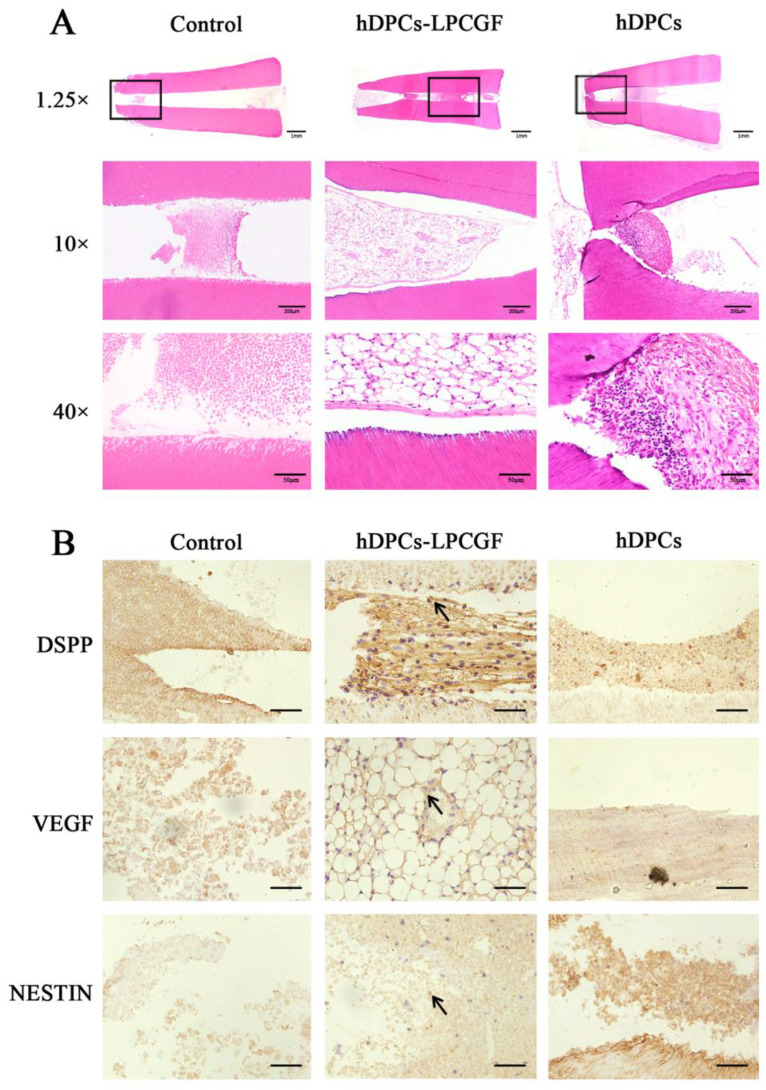
The histologic results of hDPCs-LPCGF complex on pulp regeneration and root/apical foramen development in vivo. (**A**) The HE staining results of the hDPCs-LPCGF, hDPCs and control groups. (**B**) The IHC staining results of DSPP, VEGF and NESTIN in the hDPCs-LPCGF, hDPCs and control groups, and the magnification is 40×. Black arrowheads indicate the intense positive staining. Scale bar = 50 μm.

HE staining (Figure 8A) showed that in hDPCs-LPCGF group, new pulp-like tissue was observed, including new dentin deposition in the root canal wall, vascular network structure and fibrous tissue in the lumen. In the hDPCs group, there was a small amount of fibrous tissue in the root canal. In the control group, the root canal was empty.

IHC staining (Figure 8B) showed that DSPP positive signals were mostly distributed near the odontoblast cell layer in the medullary cavity, indicating the presence of newly generated dentin. VEGF positive signals were distributed around the vessel wall, suggesting neovascularization tissue. NESTIN positive signals were scattered among the tissue in the medullary cavity, suggesting the formation of nerve-like tissue.

## 4. Discussion

The preparation of CGF and LPCGF does not require the addition of chemical activators, and different centrifugation rates in the program can be set to allow platelets to collide more adequately, thus releasing more growth factors. CGF contains abundant growth factors, including TGF-β, VEGF, PDGF, bFGF, BMP, insulin-like growth factor (IGF), epidermal growth factor (EGF) and osteoprotegerin (OPG) [24]. CGF also has better biocompatibility and more stable fibrin network structure with excellent mechanical properties of tensile strength and fracture strain, which can avoid the enzymatic hydrolysis of growth factors in the form of chemical bonds and extend its action time [32,33,34]. Furthermore, CGF contains a denser and richer GF–fibrin matrix in which growth factors are closely bound to one another [23]. This provides the slow release of growth factors, which helps with wound healing [20,35].

As a product of improved CGF technology, LPCGF exhibited positive promoting effects on hDPC proliferation, migration and dentinogenic differentiation. The results of our study showed that LPCGF promoted the proliferation of hDPCs in a concentration- and time-dependent manner, and enhanced the migration of hDPCs. It was possibly because LPCGF contained a large number of growth factors, such as BMP-2, bFGF, VEGF and PDGF, which were proved to promote the migration, proliferation and angiogenic differentiation of hDPSCs [36,37,38,39,40]. BMP-2 is a powerful osteoinductive cytokine which can induce the differentiation of undifferentiated mesenchymal cells into cartilage and new bone. The fusion of growth factor BMP-2 into the tissue engineering scaffold structure can not only improve the interface contact between hDPSCs and the scaffold, but also make the chemical composition of the material similar to the extracellular matrix, which is conducive to the growth of hDPSCs [40]. FGF-2 could activate HDPC growth and migration [36], and has an anti-apoptotic effect on fibroblasts [41]. VEGF and FGF-2 were both proved to enhance dental pulp regeneration and neovascularization [37,38]. PDGF is an effective growth factor for wound healing [39] and has the effect of stabilizing neovascularization [42]. PDGF can also promote the proliferation and recruitment of periodontal osteocytes and achieve periodontal regeneration [43]. It has also been proved that PDGF could significantly enhance hDPSC proliferation [44].

In addition, our study indicated that LPCGF could promote the dentinogenic differentiation of hDPCs. TGF-β can promote the differentiation of hDPCs into odontoblast stem cells and up-regulate the matrix secretion of the dentin-pulp complex [45]. TGF-β can also significantly increase the proliferation and ALPase activity of human dental pulp fibroblasts, which could then aggregate to form mineralization nodules [46]. BMP-7 has been reported to promote the odontogenic differentiation and mineralization of hDPSCs [47]. Interestingly, LPCGF induced the DSPP expression of hDPCs, while it had no effect on Runx2 expression. DSPP is positively expressed in the early odontoblastic differentiation of hDPSCs [48], and Runx2 is an early regulator of osteoblast differentiation [49], which indicated that under the stimulation of LPCGF, the hDPCs were more inclined to odontogenic differentiation rather than osteogenic differentiation, and made it a more suitable material for dentin-pulp complex regeneration.

BMP-2, bFGF, VEGF and PDGF-BB were quantitatively detected to investigate the difference in the release concentration in CGF and heat-treated LPCGF, and the results confirmed that both CGF and LPCGF contained these growth factors. Our results have also suggected that 25% LPCGF promoted hDPC mineralization most significantly. Therefore, although LPCGF contained less growth factors than CGF, it might play a better role in promoting odontogenic differentiation of hDPCs. And the slow release and stable concentration range of growth factors of LPCGF were superior to CGF, which ensured that sufficient local concentration of growth factors could be obtained in the following heterotopic transplantation of hDPCs-LPCGF complex in vivo.

Autologous dental pulp stem cells have great odontogenic differentiation potential, convenient access and extensive sources [7], which have a good research prospect in dental pulp regeneration through autologous stem cell transplantation. A clinical study has demonstrated the therapeutic potential of mobilized dental pulp stem cells for complete pulp regeneration [11]. Our study further revealed the potential mechanism of hDPC transplantation achieving pulp regeneration. Our results indicated that the hDPCs-LPCGF complex could promote the deposition of mineralization tissue in the root canal wall in vivo, and might continuously thicken the dentin wall after implantation. And the histologic results showed that the hDPCs-LPCGF complex could successfully regenerate dentin-pulp complex structure heterotropically. The regenerated pulp tissue, including neovascularization and nerve-like tissue, almost filled the middle and apical part of the root canal, and there was new secondary dentin deposition in the root canal wall. In hDPCs group, there was only a small amount of newly formed pulp tissue in the root canal, probably because the cell suspension was fluid without scaffold structure, thus it could not be anastomotic with host blood vessels to obtain sufficient nutritional supply, resulting in the death of the implanted stem cells [50]. The heating treatment of LPCGF turned it into a gelatinous structure, which provided a more stable scaffold for cell colonization and growth. In addition, LPCGF is easier to fully mix and contact with stem cells compared to CGF.

Overall, our research showed that The co-culture system of hDPCs and LPCGF could form a stable three-dimensional porous structure containing platelets, white blood cells and growth factors, which could make hDPCs easier to migration, adhesion, proliferation and differentiation, and the hDPCs were more inclined to odontogenic differentiation which was more suitable for dentin-pulp complex regeneration; the hDPCs-LPCGF complex is holo autogenous, and the LPCGF is degradable and can be gradually replaced by regenerated pulp tissue without residual exogenous tissue; the LPCGF can provide and persistently release various growth factors and maintain relatively stable growth factor concentration in the local microenvironment for dental pulp regeneration. Therefore, hDPCs-LPCGF complex is expected to be a feasible method for the study of pulp regeneration therapy.

## 5. Conclusions

In summary, our in vitro results shed light on the potential of LPCGF on promoting the proliferation, migration and differentiation of hDPCs, and the in vivo results proved that hDPCs-LPCGF complex could heterotropically regenerate dental pulp tissue with newly formed dentin, neovascularization and nerve-like tissue, which provides a potential in vivo mechanism of the hDPCs-LPCGF complex transplantation promoting dental pulp regeneration. However, due to the fact that LPCGF is a mixture with fibrin, growth factors, and various cells, future research may focus on the impact of a single component of LPCGF on the regeneration of the pulp-dentin complex, thereby further revealing the potential mechanism. At the same time, more randomized clinical controlled trials are needed to verify the role of hDPCs-LPCGF complex transplantation in regenerative endodontic therapy of pulpitis and periapical periodontitis of young permanent teeth.

## 6. Patents

The preparation method of hDPCs-LPCGF complex resulting from the work reported in this manuscript has been authorized for invention patent (Patent Number: CN113975467A).

## Figures and Tables

**Table 1:** Primer sequences used in the real-time PCR.

Gene	Primer Sequences
GAPDH-HUMAN-F	GGAGCGAGATCCCTCCAAAAT
GAPDH-HUMAN-R	GGCTGTTGTCATACTTCTCATGG
DSPP-F	TTTGGGCAGTAGCATGGGC
DSPP-R	CCATCTTGGGTATTCTCTTGCCT
RUNX2-F	CCTTTACTTACACCCCGCCA
RUNX2-R	GGATCCTGACGAAGTGCCAT

## Data Availability

The datasets used and/or analysed during the current study are available from the corresponding author on reasonable request.
